# Spine Injuries Sustained After Falls While Crossing the U.S.-Mexico Border

**DOI:** 10.1089/neur.2024.0035

**Published:** 2024-04-10

**Authors:** Hannah R. Riva, Michael M. Polmear, Cyrena Petersen, June Y. Guillet, Taylor M. Yong, Adam H. Adler, Rajiv Rajani, Vishwajeet Singh, David Chin Sing Wang

**Affiliations:** ^1^ Paul L. Foster School of Medicine, El Paso, Texas, USA.; ^2^ Division of Orthopaedic Surgery, El Paso, Texas, USA.; ^3^ Division of Neurosurgery, University Medical Center, El Paso, Texas, USA.; ^4^ Biostatistics and Epidemiology Consulting Lab; Texas Tech University Health Sciences Center, El Paso, Texas, USA.

**Keywords:** border, fall, spine, surgery, trauma, wall

## Abstract

This study is to report the demographics, incidence, and patterns of spinal injuries associated with border crossings resulting from a fall from a significant height. A retrospective cohort study was performed at a Level I trauma center from January 2016 to December 2021 to identify all patients who fell from a significant height while traversing the U.S.-Mexico border and were subsequently admitted. A total of 448 patients were identified. Of the 448 patients, 117 (26.2%) had spine injuries and 39 (33.3%) underwent operative fixation. Females had a significantly higher incidence of spine injuries (60% vs. 40%; *p* < 0.00330). Patients with a spine fracture fell from a higher median fall height (6.1 vs. 4.6 m; *p* < 0.001), which resulted in longer median length of stay (LOS; 12 vs. 7 days; *p* < 0.001), greater median Injury Severity Score (ISS; 20 vs. 9; *p* < 0.001), and greater relative risk (RR) of ISS >15 (RR = 3.2; *p* < 0.001). Patients with operative spine injuries had significantly longer median intensive care unit (ICU) LOS than patients with non-operative spine injuries (4 vs. 2 days; *p* < 0.001). Patients with spinal cord injuries and ISS >15 sustained falls from a higher distance (median 6.1 vs. 5.5 m) and had a longer length of ICU stay (median 3 vs. 0 days). All patients with operative spine injuries had an ISS >15 relative to 50% of patients with non-operative spine injuries (median ISS 20 vs. 15; *p* < 0.001). Patients with spine trauma requiring surgery had a higher incidence of head (RR = 3.5; *p* 0.0353) and chest injuries (RR = 6.0; *p* = 0.0238), but a lower incidence of lower extremity injuries (RR = 0.5; *p* < 0.001). Thoracolumbar injuries occurred in 68.4% of all patients with spine injuries. Patients with operative spine injuries had a higher incidence of burst fracture (RR = 15.5; *p* < 0.001) and flexion-distraction injury (RR = 25.7; *p* = 0.0257). All patients with non-operative spine injuries had American Spinal Injury Association (ASIA) D or E presentations, and patients with operative spine injuries had a higher incidence of spinal cord injury: ASIA D or lower at time of presentation (RR = 6.3; *p* < 0.001). Falls from walls in border crossings result in significant injuries to the head, spine, long bones, and body, resulting in polytrauma casualties. Falls from higher height were associated with a higher frequency and severity of spinal injuries, greater ISS, and longer ICU length of stay. Operative spine injuries, compared with non-operative spine injuries, had longer ICU length of stay, greater ISS, and different fracture morphology. Spine surgeons and neurocritical care teams should be prepared to care for injuries associated with falls from height in this unique population.

## Introduction

Injuries sustained crossing the U.S.-Mexico border have changed with immigration policies and barriers with falls from height increasing in recent years.^1–4^ Our institution in El Paso, Texas, is the only Level I trauma center in a 280-mile radius along the U.S.-Mexico border and has observed an increase in patients sustaining falls and injuries traversing the border that require admission and operative management since 2017.^5^ The purpose of this study is to report the demographics, incidence, and patterns of spine and concomitant injuries associated with border crossings resulting in fall from height.

**FIG. 1.**
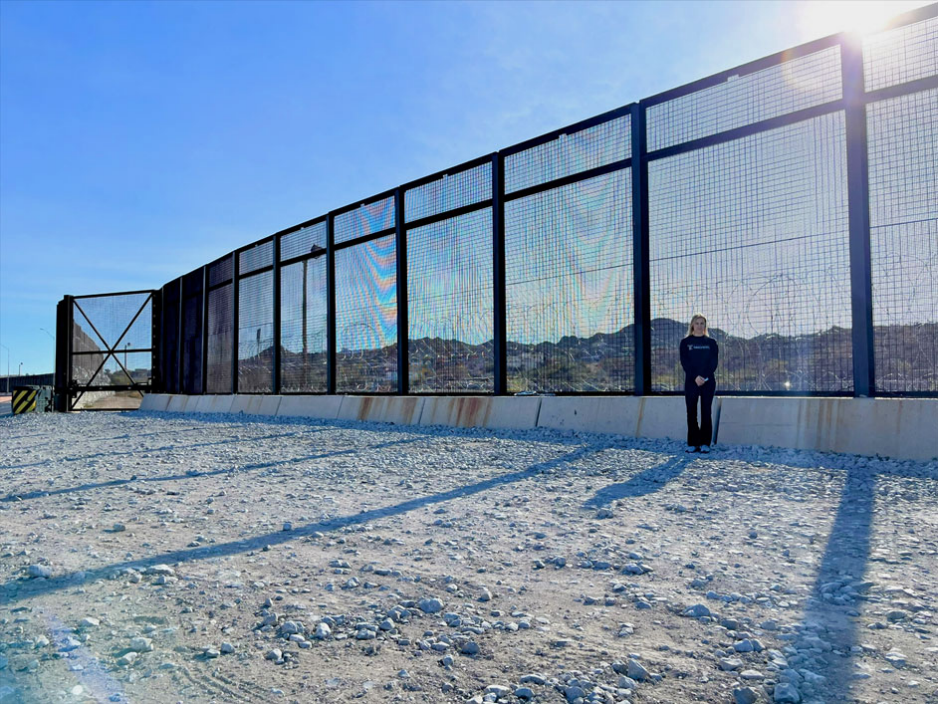
The steel bollard U.S.-Mexico barrier may be observed, here demonstrating the barrier at 5.5 m (18 ft) in height; the barrier in other sites along the border has been extended to 9.1 m (30 ft) in height.

Falls from height result most commonly in lower extremity fractures followed in frequency by thoracolumbar spine fractures.^4,6,7^ A 2017 study found a significant association between thoracolumbar fractures, calcaneal fractures, and tibial pilon fractures, supporting the mechanism of axial force transmission from the appendicular skeleton to the spinal column.^7^ Injuries associated with falls from height depend on several factors, including fall height, accidental versus non-accidental fall pattern influencing fall orientation and mechanism of landing, and landing surface.^8,9^ The mechanism of each fall in patients from the border barrier is variable, based on use of climbing aids and/or attempts to downclimb, and may differ from previous reports on intentional jumping, given that human smugglers may push or intentionally drop fence-crossers in order to flee border patrol agents.^7^ The steel bollard barrier may be observed in the photo in Figure 1, which shows the barrier at 5.5 m (18 ft) in height; the barrier in other sites along the border has been extended to 9.1 m (30 ft) in height. Previous work has demonstrated that U.S. Border Patrol (USBP) apprehensions correlate with hospital admissions, suggesting that a consistent proportion of migrants sustain injuries in traversing the border.^5,10^

By an executive order in 2017, 406 miles of previous border wall barriers from 6 to 17 ft along the U.S.-Mexico border were replaced with a 30-ft-tall steel barrier, and an additional 49 miles of new barrier were added. Liepert and colleagues in 2022 reported significantly increased migrant deaths, trauma center admissions, and injury severity at the University of California San Diego Level I trauma center after the increase in border wall height.^10^

Musculoskeletal fractures are described as the most common injury type in border crossing injuries sustained.^11,12^ In a study evaluating patients from 2013 to 2019 at the California-Baja border, the most common injuries sustained were spine, pilon, and calcaneus fractures.^13^

A study examining neurotrauma in patients in border wall injuries from 2012 to 2017 at Banner University of Arizona Medical Center in Tucson, Arizona found that spine injuries comprised 78% of neurotrauma injuries sustained; burst fractures were the most common spine fracture followed by compression fractures, most frequently occurring in the lumbar spine.^14^ The increased frequency of spine fractures specifically at the thoracolumbar junction would be expected with increased fall height based on amplified force from higher fall heights.^9,15^ A recent study showed that the San Diego-Mexico border wall height extension correlated with more frequent, severe, and costly spinal injuries.^16^

Injury Severity Score (ISS) assesses injuries in nine body regions from the Abbreviated Injury Scale (AIS) by grouping the regions into six: head or neck; face; chest; abdominal or pelvic contents; extremities or pelvic girdle; and external. ISS is calculated by taking the sum of the squares of the highest AIS scores of the three body regions with the most severe injuries.^17^ The New Injury Severity Score (NISS) considers the three most severe injuries regardless of body region and has been shown in some studies to more accurately predict mortality, ICU admission, and ICU length of stay.^17,18^ We use these measures to classify severity of injury attributable to fall from border wall.

## Methods

The trauma registry at a Level I trauma center was queried from January 2016 through December 2021 to identify all patients who were injured in falls from height while crossing the U.S.-Mexico border and who presented with injuries requiring admission. An analysis was performed to assess the types of injuries identified and the surgical interventions. A cohort analysis was performed among patients admitted with spine injuries with a subgroup analysis between spine injuries treated non-operatively and operatively. The primary outcome variable was ISS. From past data, a power analysis was performed for comparing ISS between patients treated non-operatively and operatively for spine injuries. A sample size of 31 patients per group was planned based on an expected effect size of 5 for the primary outcome measure of ISS,^19^ calculated to yield 80% power to detect a different median difference using the Mann-Whitney U test with a *p* value of <0.05. The registry query dates provided a sample size of 117 patients with spine injuries sustained from traversing the U.S.-Mexico border, of which 39 were treated operatively. All patients had sufficient registry data to be included in the analysis.

All patients admitted under USBP custody crossing the border were included. Patients who were admitted under USBP custody with mechanisms other than fall from border wall (e.g., motor vehicle accidents, pedestrian accidents, and exposure) were excluded because of the absence of consistent corroboration of a fall from height and to minimize heterogeneity.

Data collected included demographics (e.g., age and sex), injury mechanism, fall height based on structure height at injury site, length of stay (LOS), intensive care unit (ICU) admission and ICU LOS, injury characteristics, operations performed, and clinical follow-up dates. ISS, American Spinal Injury Association (ASIA) Impairment Scale, and Thoracolumbar Injury Classification and Severity (TLICS) Scale were calculated for each applicable case.

### Statistical analysis

Calculations were performed utilizing SAS software (SAS Institute Inc., Cary, SC). Normality was assessed with the Shapiro-Wilk expanded test. Continuous variables were assigned using median and interquartile range (IQR), and categorical variables were described using frequency and percentage. Medians between groups of continuous non-parametric data were compared using the Mann-Whitney U test. For binary outcome cohort comparisons, relative risk (RR) and 95% confidence intervals (CIs) were calculated. To assess correlation, linear regression analysis was conducted. Statistical significance was defined at an alpha value <0.05 or with Bonferroni's correction for multiple comparisons.

## Results

A total of 448 patients with falls from height while crossing the U.S.-Mexico border were identified from 2016 to 2021 (Table 1). There was a significant increase in all hospital admissions subsequent to falls from the international border throughout the study time period. Similarly, the incidence of patients admitted with non-operative and operative spine injuries increased significantly and was correlated with total number of patients admitted with injuries sustained falling from height while traversing the border.

**Table 1.** Annual Admissions by Number of Patients With Non-Operative and Operative Spine Injuries

***Year***	***All***	***Spine injury***		***Non-operative spine***			***Operative spine***		
	*N*	*N*	***% All***	*N*	***% All***	***% Spine***	*N*	***% All***	***% Spine***
2016	3	0	0.0	0	0.0	0.0	0	0.0	0.0
2017	10	1	10.0	1	10.0	100.0	0	0.0	0.0
2018	16	3	18.8	3	18.8	100.0	0	0.0	0.0
2019	91	21	23.1	15	16.5	71.4	6	6.6	28.6
2020	104	30	28.8	17	16.3	56.7	13	12.5	43.3
2021	224	62	27.7	42	18.8	67.7	20	8.9	32.3
*Total*	*448*	*117*	*26.1*	*78*	*17.4*	*66.7*	*39*	*8.7*	*33.3*
Correlation coefficient (*p* value)	0.919 (<0.001)	0.997 (<0.001)		0.998 (<0.001)		1.0 (<0.001)	0.972 (0.00118)		0.983 (<0.001)

Of the 448 patients, 117 (26.2%) persons had spine injuries. There were statistically significant differences in patients sustaining spine injuries (Table 2). Females sustained a significantly higher incidence of spine injuries relative to males with an RR of 1.61 (95% CI, 1.2–2.2; *p* = 0.00330). There was no significant difference in incidence of operative spine injuries between females and males (RR = 1.0; 95% CI, 0.8–2.3; *p* = 0.297). Patients with spine injuries had a significantly longer median hospital LOS (12 [IQR 10] vs. 7 [IQR 10] days; *p* < 0.001). There was no significant difference in median hospital LOS between patients with operative and non-operative spine injuries (12 [IQR 10] vs. 11.5 [IQR 10] days; *p* = 0.090).

**Table 2.** Demographics With Median Values for All Patients by Operative Treatment of Spine Injury

***Factor***	***All***	***Spine injury***			***Spine injury,*** *N* *** = 117***		
		***No***	***Yes***	*p* ***value***	***Non-operative***	***Operative***	*p* ***value***
*N* (%)	448	331 (73.9)	117 (26.1)	—	78 (66.7)	39 (33.3)	—
Age (years, IQR)	30.0 (16)	30.0 (16)	29.0 (17)	0.56	30.0 (17)	28.0 (16)	0.67
Sex, *N* (%)							
Female	215 (48.0)	145 (43.8)	70 (59.8)	RR = 1.6 CI = 1.2–2.2 *p* = 0.00330	44 (56.4)	26 (66.7)	RR = 1.0 CI = 0.8–2.3 *p* = 0.30
Male	233 (52.0)	186 (56.2)	47 (40.2)		34 (43.6)	13 (33.3)	
BMI (IQR)	28.5 (4.8)	28.4 (4.9)	29.0 (4.6)	0.64	28.8 (4.5)	29.1 (4.8)	0.71
Hospital LOS (days, IQR)	8.0 (10)	7.0 (10)	12.0 (10)	<0.001	11.5 (10)	12.0 (10)	0.09
ICU LOS (days, IQR)	0.0 (1.5)	0.0 (0)	2.0 (4)	<0.001	2.0 (3)	4.0 (2)	<0.001
Distance (m, IQR)	5.0 (3.1)	4.6 (3.1)	6.1 (1.1)	<0.001	6.0 (1.1)	6.10 (3.1)	0.30
≥5 m, *N* (%)	242 (54.0)	154 (46.5)	88 (75.2)	RR = 2.6 CI = 1.7–3.9 *p* < 0.001	59 (75.6)	29 (74.4)	RR = 1.0 CI = 0.8–1.2 *p* = 0.88
ISS (IQR)	9.0 (7)	9.0 (5)	20.0 (11)	<0.001	15.0 (7)	20.0 (5)	<0.001
ISS >15, *N* (%)	148 (33.0)	70 (47.3)	78 (52.7)	RR = 3.2 CI = 2.5–4.0 *p* < 0.001	39 (50.0)	39 (100)	RR = 3.0 CI = 2.3–3.9 *p* < 0.001

BMI, body mass index (kg/m^2^); CI, 95% confidence interval; ICU, intensive care unit; IQR, interquartile range; LOS, length of stay; m, meters; ISS, Injury Severity Score; RR, relative risk.

Patients with spine injuries had significantly longer median ICU LOS (2 [IQR 4] vs. 0 [IQR 0] days; *p* < 0.001). Patients with operative spine injuries had significantly longer median ICU LOS than patients with non-operative spine injuries (4 [IQR 2] vs. 2 [IQR 3] days; *p* < 0.001). Patients with a spine injury had significantly higher falls (6.1 [IQR 1] vs. 4.6 [IQR 3.1] m; *p* < 0.001), with an increased RR of sustaining a fall ≥5 m (RR = 2.6; 95% CI, 1.7–3.9; *p* < 0.001). There was no significant difference in fall height between patients with operative and non-operative spine injuries (*p* = 0.30) nor association with falling ≥5 m (RR = 1.0; 95% CI, 0.8–1.2; *p* = 0.88). Patients with spine injuries had significantly higher median ISS (20 [IQR 11] vs. 9 [IQR 5]; *p* < 0.001) and greater RR of ISS >15 (RR = 3.2; 95% CI, 2.5–4.0; *p* < 0.001). All patients with operative spine injuries had an ISS >15 relative to 50% of patients with non-operative spine injuries (median ISS 20 [IQR 5] vs. 15 [IQR 7]; *p* < 0.001; RR = 3.0; 95% CI, 2.3–3.9; *p* < 0.001).

Patients with spine injuries and ISS >15 sustained falls from a higher distance (median 6.1 [IQR 2.1] vs. 5.5 [IQR 2.1] m) and had a longer length of ICU stay (median 3 [IQR 2] vs. 0 [IQR 0] days; Table 3).

**Table 3.** Distribution of Demographic and Injury Variables by ISS Category (≤15 vs. >15) in Patients With Spine Injuries

***Factor***	***Total***	***ISS ≤15***	***ISS >15***	*p* ***value***
*N*	117	39	78	
Age, median (IQR)	29.0 (17)	29.00 (17)	29.50 (18)	0.21
Sex (%)				0.37
Female	70 (59.8)	21 (53.9)	49 (62.8)	
Male	47 (40.2)	18 (46.2)	29 (37.2)	
BMI, median (IQR)	28.6 (6.3)	28.4 (5.2)	28.7 (7.0)	0.94
Distance (m), median (IQR)	6.1 (1.1)	5.5 (2.1)	6.1 (2.1)	0.0140
Hospital LOS (days), median (IQR)	12.0 (10)	11.0 (8)	12.0 (12)	0.16
ICU LOS (days), median (IQR)	2.0 (4)	0 (0)	3.0 (2)	<0.001

BMI, body mass index (kg/m^2^); ICU, intensive care unit; IQR, interquartile range; LOS, length of stay (days); m, meters; ISS, Injury Severity Score.

Concomitant injuries within the ISS body regions occurred at different frequencies and operative rate between patients with operative and non-operative spine injuries (Table 4). Patients with operative spine injuries had a higher incidence of head (RR = 3.5; 95% CI, 1.1–11.2; *p* = 0.0353) and chest injuries (RR = 6.0; 95% CI, 1.3–28.4; *p* = 0.0238). In contrast, patients with operative spine injuries had a lower incidence of lower extremity injuries (RR = 0.5; 95% CI, 0.4–0.8; *p* < 0.001). Operative frequencies of concomitant injuries followed a similar pattern, with patients with operative spine injuries undergoing lower extremity operations less frequently (RR = 0.4; 95% CI, 0.2–0.6; *p* < 0.001).

**Table 4.** Injury Variables of Patients by Operative Treatment of Spine Injury

***Factor***	***Spine***			***Non-operative spine***			***Operative spine***			***Relative risk^a^ [95% CI]***	*p* ***value***
	*N* *** = 117***	***% All*** *N* *** = 448***	***% Spine*** *N* *** = 117***	*N* *** = 78***	***% Spine*** *N* *** = 117***	***% Non-Op*** *N* *** = 78***	*N* *** = 39***	***% Spine*** *N* *** = 117***	***% Op*** *N* *** = 39***		
		***26.1***	***100***		***66.7***	***100***		***33.3***	***100***	***—***	
Concomitant non-spine injury											
Head	11	2.5	9.4	4	3.4	5.1	7	6.0	17.9	3.5 [1.1–11.2]	0.0353
Face	6	1.3	5.1	3	2.6	3.8	3	2.6	7.7	2.0 [0.4–9.5]	0.38
Chest	8	1.8	6.8	2	1.7	2.6	6	5.1	15.4	6.0 [1.3–28.4]	0.0238
Abdomen	7	1.6	6.0	5	4.3	6.4	2	1.7	5.1	0.8 [0.2–3.9]	0.78
Upper extremity	20	4.5	17.1	14	12.0	17.9	6	5.1	15.4	0.9 [0.4–2.1]	0.73
Pelvic	32	7.1	27.4	21	17.9	26.9	11	9.4	28.2	1.0 [0.6–1.9]	0.88
Lower extremity	82	18.3	70.1	65	55.6	83.3	17	14.5	43.6	0.5 [0.4–0.8]	<0.001
Non-spine surgery											
Head/neck, face, chest, abdomen	9	2.0	7.7	5	4.3	6.4	4	3.4	10.3	1.6 [0.5–5.6]	0.46
Upper extremity	19	4.2	16.2	16	13.7	20.5	3	2.6	7.7	0.4 [0.1–1.2]	0.10
Pelvic	11	2.5	9.4	9	7.7	11.5	2	1.7	5.1	0.5 [0.1–2.0]	0.30
Lower extremity	90	20.1	76.9	76	65.0	97.4	14	12.0	35.9	0.4 [0.2–0.6]	<0.001
Major spine injury region											
Cervical	1	0.2	0.9	1	0.9	1.3	0	0.0	0.0	0.7 [0.03–15.8]	0.80
Thoracic (T1–T9)	9	2.0	7.7	5	4.3	6.4	4	3.4	10.3	1.6 [0.5–5.6]	0.46
Thoracolumbar (T10–L2)	80	17.9	68.4	52	44.4	66.7	28	23.9	71.8	1.1 [0.8–1.4]	0.56
Lumbar (L3–L5)	27	6.0	23.1	20	17.1	25.6	7	6.0	17.9	0.7 [0.3–1.5]	0.36
Major spine injury fracture morphology											
Burst	35	7.8	29.9	4	3.4	5.1	31	26.5	79.5	15.5 [5.9–40.8]	<0.001
Compression	60	13.4	51.3	59	50.4	75.6	1	0.9	2.6	0.03 [0.004–0.2]	<0.001
Compression >20%	5	1.1	4.3	4	3.4	5.1	1	0.9	2.6	0.5 [0.06–4.3]	0.53
Flexion-distraction	6	1.3	5.1	0	0.0	0.0	6	5.1	15.4	25.7 [1.5–444]	0.0257
Spinous process	1	0.2	0.9	1	0.9	1.3	0	0.0	0.0	0.6 [0.03–15.8]	0.80
Transverse process	10	2.2	8.5	10	8.5	12.8	0	0.0	0.0	0.09 [0.006–1.6]	0.1
Multiple spine fractures											
1	60	13.4	51.3	42	35.9	53.8	18	15.4	46.2	0.9 [0.6–1.4]	0.66
2	35	7.8	29.9	17	14.5	21.8	18	15.4	46.2	1.8 [1.0–3.1]	0.0532
3	12	2.7	10.3	7	6.0	9.0	5	4.3	12.8	1.4 [0.5–4.1]	0.56
4	9	2.0	7.7	7	6.0	9.0	2	1.7	5.1	0.6 [0.1–2.7]	0.50
5	5	1.1	4.3	4	3.4	5.1	1	0.9	2.6	0.5 [0.06–4.4]	0.54
6	3	0.7	2.6	0	0	0	3	2.6	7.7	12.9 [0.7–243]	0.089
7	1	0.2	0.9	1	0.9	1.3	0	0.0	0	0.7 [0.03–16]	0.80
TLICS											
1	61	13.6	52.1	61	52.1	78.2	0	0.0	0.0	0.03 [0.002–0.5]	0.0115
2	12	2.7	10.3	12	10.3	15.4	0	0.0	0.0	0.09 [0.006–1.5]	0.094
3	3	0.7	2.6	4	2.6	3.8	0	0.0	0.0	0.3 [0.01–4.2]	0.32
4	4	0.9	3.4	1	0.9	1.3	3	2.6	7.7	5.6 [0.6–52.6]	0.13
5	11	2.5	9.4	0	0.9	1.3	10	8.5	25.6	33.2 [2.0–553]	0.00710
6	2	0.4	1.7	0	0.0	0.0	2	1.7	5.1	9.4 [0.5–191]	0.14
7	10	2.2	8.5	0	0.0	0.0	10	8.5	25.6	33.2 [2.0–554]	0.0148
8	10	2.2	8.5	0	0.0	0.0	10	8.5	25.6	33.2 [2.0–554]	0.0148
9	1	0.2	0.9	0	0.0	0.0	1	0.9	2.6	5.8 [0.2–139]	0.28
10	3	0.7	2.6	0	0.0	0.0	3	2.6	7.7	12.9 [0.7–243]	0.089
ASIA											
A	2	0.4	1.7	0	0.0	0.0	2	1.7	5.1	9.9 [0.5–200]	0.14
B	2	0.4	1.7	0	0.0	0.0	2	1.7	5.1	9.9 [0.5–200]	0.14
C	1	0.2	0.9	0	0.0	0.0	1	0.9	2.6	5.9 [0.2–142]	0.27
D	25	5.6	21.4	6	5.1	7.7	19	16.2	48.7	6.3 [2.8–14.6]	<0.001
E	87	19.4	74.4	72	61.5	92.3	15	12.8	38.5	0.8 [0.6–1.1]	0.20

^a^ Relative risk between patients with non-operative and operative spine.

ASIA, American Spinal Injury Association Impairment Scale; CI, confidence interval; IQR, interquartile range; Non-Op, non-operative; Op, operative; TLICS, Thoracolumbar Injury Classification and Severity Scale.

Major spine injury region (cervical, thoracic, thoracolumbar, and lumbar) incidence distribution was similar between the two groups (Table 4). Thoracolumbar injuries had 17.9% incidence in all patients admitted and constituted the majority in each group, occurring in 68.4% of all patients with spine injuries, 66.7% treated non-operatively, and 71.8% treated operatively. In addition to major spine fracture morphologies, 65 patients (55.6% of patients with spine fractures) sustained at least two fractures. There was no difference in the incidences of multiple spine fractures between patients treated operatively and non-operatively (RR ≥2 fractures; 1.4 [0.9–2.0]; *p* = 0.13).

However, there were differences in spine injury morphology between the groups. Patients with operative spine injuries had a lower incidence of compression fractures (RR = 0.3; 95% CI, 0.004–0.2; *p* < 0.001) and higher incidences of burst (RR = 15.5; 95% CI, 5.9–40.8; *p* < 0.001) and flexion-distraction (RR = 25.7; 95% CI, 1.45–444; *p* = 0.0257). Patients with operative spine injuries had a higher TLICS score (median 7 [IQR 3] operative vs. 1 [IQR 1] non-operative, median 1 [IQR 4] all; *p* < 0.001). In the non-operative spine group, all patients had TLICS score ≤3, except for 1 patient with an L4 burst fracture and pilon fracture without neurological symptoms (TLICS 4), who was treated with bracing. All patients with non-operative spine injuries had ASIA D or E presentations, and patients with operative spine injuries had a higher incidence of ASIA D presentation (RR = 6.3; 95% CI, 2.8–14.6; *p* < 0.001).

### Operative management

Characteristics of the 39 operative spine patients are included in Supplementary Table S1. Spine injuries occurred in 117 (26.1%) patients, of which 39 underwent operative treatment, constituting a 33.3% operative rate for patients with spine injuries and 8.7% of all patients.

Patients with spine injuries treated operatively were treated with decompression and instrumented stabilization within a median of 1 day from presentation (IQR 1, range 0–13 days). Three patients (7.7%) treated operatively for spine injuries also underwent one other non-musculoskeletal surgery, including a cholecystectomy, craniotomy, and scalp laceration repair. There was one complication in a patient treated operatively for a spinal injury that necessitated revision.

### Follow-up

Follow-up in affiliated clinics occurred in 6 patients with spine injuries treated operatively (15.4%) with a median duration of 21 days (IQR 23 days, range 13–47). Fifty-five patients (12.2%) with musculoskeletal injuries had a median clinic follow-up duration of 28 days (IQR 36 days, range 6 days to 8 months) in affiliated local clinics. Patients evaluated in the clinic were pending transfer disposition or were staying with local family members. Barriers to care included access to durable medical equipment for adherence to weight-bearing and bracing restrictions, discharge instruction translation into native language, suture and staple removal with wound evaluation, and medication refills.

## Discussion

After lower extremity fractures, thoracolumbar fractures are the next most common injury in border crossing falls; thus, spine injuries in this population merit investigation. As anticipated, increased barrier height at the scene of injury was associated with higher ISS, increased the relative risk of ISS >15, and increased the risk of spine injury. Also, patients with a spine injury had higher height from fall, longer LOS, greater ISS, and greater RR of ISS >15. The distance fallen was the primary demographic and injury variable that was significantly associated with ISS >15, a commonly recognized value in defining polytrauma as well as the interchangeably used terms severely injured and major trauma. Distance fallen has been shown to be an independent predictor of trauma severity, and this was redemonstrated in our study.^20,21^

Further, the mechanics of border wall falls can differ from those of domestic falls. Falling from the border wall may result in variable injury patterns attributable to intention, accident, and use of climbing aides. Migrants may intentionally jump in order to flee, resulting in an increased bracing for the fall associated with intentional falls. Bracing for the fall may lead to increased axial loading, and these factors have been described as resulting in an increased rate of extremity and spine injuries in border wall falls.^22^ Accidental falls from climbing aides may limit bracing and result in more variable injury patterns than those observed in the intentional fall mechanisms.

Females made up a significantly higher portion of spine injuries than of overall injuries, and females had significantly more spine injuries than males in our study population. Reasons for this finding have not been clearly elucidated and warrant further investigation, given that this represents a disparity in this population. A recent study on spine injuries from the San Diego-Mexico border wall did not show a significant difference between males and females with respect to incidence of spine injuries in their study population; however, comparisons between numbers of patients with and without spine injuries were not reported.^16^

Patients with spine injuries treated operatively underwent lower extremity fracture fixation operations less frequently than did patients with spine injuries treated non-operatively. One hypothesis for this is differences in the fall mechanics and energy absorption between the two groups of patients with spine injuries. These patients treated operatively for their spine fractures also had significantly higher incidences of head and chest injuries, as well as burst fracture morphology patterns, suggesting energy absorption to the axial, rather than the appendicular, skeleton. Spine fractures in falls from height are often associated with lower extremity fractures.^7,23,24^ The finding that operative spine injuries were less associated with surgery for lower extremity fractures than were non-operative spine injuries represents a potential nuance in our population of border wall falls and injury patterns that has not been described yet in the literature.

Increasing border wall height has implications for resulting injuries in terms of patient disability and federal taxpayer dollars. Cost and policy analyses are beyond the scope of this article, which seeks to define the differences in injury patterns between patients with spine injuries treated non-operatively and operatively. However, it would be incomplete not to acknowledge that patient disability, rehabilitation, cost, disposition, and association with policy are critical sequelae and factors inherent to the issue of migration, border wall crossings, and resulting injuries.

**FIG. 2.**
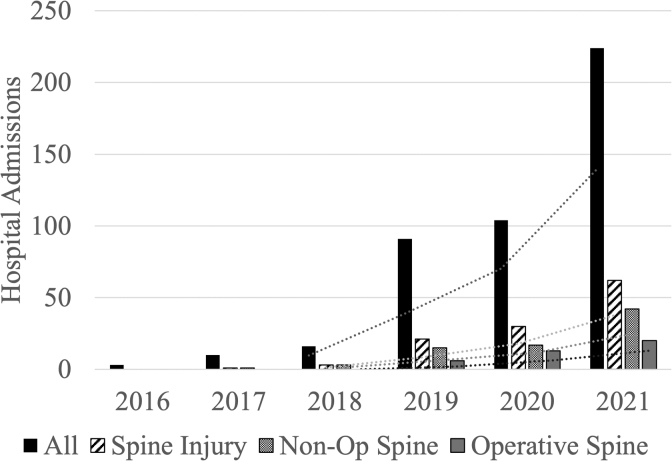
Number of hospital admissions by presence and operative treatment of spine injury throughout the study period from January 2016 through December 2021. Trendlines are 3-year moving averages.

A recent analysis of charges and costs of orthopedic injuries sustained in border wall crossings admitted to our Level I trauma center from 2017 to 2019 showed a per patient value of $121,570 with a total value of $13.5 million for 111 patients.^14^ Further, annual charges increased proportionately with increased admissions numbers over the years (e.g., $1.2 million in 2017 to $10.3 million in 2019).^25^ Numbers of admissions for border wall crossing injuries have further increased significantly in our center since 2019 (Fig. 2), and costs would be expected to continue to increase in proportion. A different study at a trauma center in southern Texas reported a reimbursement of only $0.99 million of $4.5 million in costs for undocumented immigrants brought to the trauma center after apprehension by border patrol from 2011 to 2014.^26^ Funding for caring for migrant populations include county, state, and federal sources.

There was difficulty associated with language barriers, unpredictable access to care, and suboptimal support systems, reflected by the only 12% and 15% affiliated clinic follow-up rates for musculoskeletal and operatively treated spine injuries, respectively. Deportation, detention, asylum within the United States, and humanitarian release to family members within the United States were the most common dispositions. The discharge to a location other than the locale of the treating trauma center is one significant reason for the dismal follow-up rate. The follow-up rates are similar, however, to follow-up trends in other at-risk populations.^25,27^

This study has limitations. First, lack of follow-up attributable to variable dispositions limits understanding of the post-surgical complication profile and precluded the ability to obtain radiographical or functional outcomes. Second, the study included a single Level I trauma center with a catchment area encompassing ∼13% of the U.S.-Mexico border, but did not include patients treated at lower acuity local centers, underestimating injury incidence within the catchment area. This study elucidated a pattern and treatment characterization of spine injuries at a Level I trauma center along the U.S.-Mexico border during a 6-year period.

## Conclusion

Injuries associated with border crossings and falls from height are severe with polytraumatized patients. Higher fall height correlated with more severe injury and increased risk for spinal injury. Females suffered a higher proportion of spinal injuries in our study, representing a sex disparity in this population. Most injuries in our study were falls from the international border wall in a non-geriatric population. Persons who crossed into the United States and sustained injuries with falls from height presented with unique care challenges. These patients can be particularly complex to treat given lack of available medical history, presence of medical comorbidities, language barriers, variable disposition, and extenuating social determinants of health. Spine surgeons as well as neurocritical care teams should be prepared to care for the types of injuries associated with falls from height in this unique population.

## Supplementary Material

Supplemental data

## Abbreviations Used

AIS: Abbreviated Injury Scale
ASIA: American Spinal Injury Association
CI: confidence interval
ICU: intensive care unit
IQR: interquartile range
ISS: Injury Severity Score
LOS: length of stay
NISS: New Injury Severity Score
RR: relative risk
TLICS: Thoracolumbar Injury Classification and Severity Scale
USBP: United States Border Patrol
