# Supplementary material for: Spine Injuries Sustained After Falls While Crossing the U.S.-Mexico Border
Source: Neurotrauma Rep. 2024 Apr 10;5(1):367–75. doi: 10.1089/neur.2024.0035 (PMC11035857; doi:10.1089/neur.2024.0035)
Supplement: Supplemental data [file Suppl_TableS1.docx]

Supplemental Table 1: Demographic and injury variables for patients with spine injuries treated operatively.

| **Age (y)** | **Sex** | **BMI**  **(kg/m^2^)** | **Comorbidities** | **Distance (m)** | **LOS** | **ICU LOS** | **Ortho**  **Injuries** | **Spine**  **Injuries** | **TLICS** | **ASIA** | **Spine Surgery** | **Other**  **Injuries** | **ISS** |
| --- | --- | --- | --- | --- | --- | --- | --- | --- | --- | --- | --- | --- | --- |
| 28 | F | 32.6 | - | 5 | 10 | 5 | Tibial plafond fx | TL: L2 comp fx >20% | 6 | E | L1-3 posterior fusion | - | 25 |
| 51 | F | 36.3 | - | 3 | 12 | 4 | Femoral proximal fx | TL: L2 flexion-distraction fx | 7 | E | T12-L4 posterior fusion | TBI | 29 |
| 25 | F | 34.9 | Pregnancy | 6 | 17 | 6 | - | T: T8 burst fx,  T7 and T9 comp fx,  T8 and T9 TP fx | 8 | A | T6-10 posterior fusion | TBI/ICH, SDH, SAH, IPH, subgaleal hematoma, skull fx, occipital and sphenoidal bone fx, facial laceration, right rib 1, 7, 8 fx, pneumothorax, pulmonary contusion, cholecystitis | 66 |
| 44 | M | 28.6 | Tobacco  HTN | 6 | 20 | 8 | Scapula fx | TL/L: L1 flexion-distraction fx,  L1-L5 TP fx | 7 | E | T11-L3 posterior fusion | TBI/ICH, SDH, EDH, SAH, IPH, occipital bone fx | 45 |
| 25 | F | 23 | - | 3 | 8 | 6 | - | TL: L2 burst fx,  L1 comp fx | 7 | D | L1-3 posterior fusion, interbody | - | 16 |
| 23 | F | 24.5 | - | 5 | 8 | 4 | - | TL: L1 burst fx | 5 | E | T11-L1 posterior fusion | - | 16 |
| 26 | F | 36.8 | - | 6 | 20 | 3 | Coccyx fx | TL: L2 burst fx,  L2 TP fx | 8 | D | T12-L4 posterior fusion, interbody | - | 20 |
| 34 | F | 22.7 | Tobacco  Pregnancy | 3 | 11 | 2 | Calcaneus fx | TL: L1 burst fx | 8 | D | T11- L3 post fusion | - | 20 |
| 22 | M | 37 | COVID-19  DM2 | 9 | 9 | 3 | - | TL: T12 burst fx | 4 | E | T9-L2 posterior fusion | TBI/ICH, SDH, SAH, subgaleal hematoma, scalp laceration, left 2-7 rib fx, sternoclavicular joint dislocation | 25 |
| 16 | M | 26.2 | COVID-19 | 9 | 2 | 3 | Radial distal fx | T: T5 burst fx,  T6 comp fx | 7 | C | T3-8 posterior fusion | Rib fx (right pneumomediastinum, left 1,2,4 rib fx, pneumothorax | 20 |
| 21 | F | 27.9 | - | 5 | 20 | 3 | Tibial plafond fx | TL: L1 and L2 comp fx | 5 | E | T11-L4 posterior fusion | - | 20 |
| 45 | F | 33.2 | - | 8 | 17 | 2 | Monteggia fx,  Calcaneus fx | L: L3 burst fx | 8 | D | L2-4 posterior fusion | - | 25 |
| 28 | F | 27.3 | - | 6 | 16 | 6 | Knee ligament injury,  Tibial plateau fx | TL: L1 burst fx | 8 | D | T12-L3 posterior fusion | TBI/ICH, SDH, left 5 rib fx, pulmonary contusion | 41 |
| 39 | F | 24 | - | 8 | 6 | 3 | Tibial plateau fx, Knee traumatic arthrotomy, Calcaneus fx, Midfoot fx, Coccyx fx | TL: L2 burst fx | 8 | D | T12-L4 posterior fusion | - | 32 |
| 35 | M | 31.5 | - | 7 | 17 | 3 | Coccyx fx | TL: L1 burst fx | 5 | E | T11-L3 posterior fusion | - | 20 |
| 35 | M | 29.4 | - | 9 | 17 | 4 | Coccyx fx | TL: L1 burst fx | 8 | E | T11-L3 posterior fusion | - | 20 |
| 25 | F | 33.6 | - | 5 | 9 | 3 | Coccyx fx | TL: L2 burst fx,  L2 TP fx | 4 | D | T12-L4 posterior fusion | Splenic laceration | 20 |
| 21 | F | 32.4 | - | 9 | 20 | 5 | Ankle traumatic arthrotomy,  Talus fx,  Ankle fx | TL/L: L4 burst fx,  T11-L3 comp fx | 8 | D | L2-S1 posterior fusion, interbody | - | 29 |
| 29 | F | 36.7 | - | 6 | 23 | 3 | Tibial plafond fx,  Posterior tibial tendon laceration,  Calcaneus fx,  Coccyx fx | TL: L1 burst fx | 5 | E | T12-L2 posterior fusion | - | 25 |
| 18 | M | 17.3 | - | 5 | 12 | 3 | - | TL/L: L1 burst fx,  T12 and L3 comp fx | 5 | E | T11-L3 posterior fusion | - | 20 |
| 19 | M | 27.5 | - | 8 | 14 | 3 | Coccyx fx,  Forefoot fx | TL/L: L1 burst fx,  L2-4 comp fx | 10 | B | T11-L3 posterior fusion | - | 20 |
| 23 | M | 24.2 | - | 6 | 11 | 3 | Radial head fx | TL: L1 burst fx | 6 | D | T11-L3 posterior fusion | - | 20 |
| 36 | M | 25 | - | 6 | 28 | 5 | - | C/T: T5 burst fx,  T6 burst fx, C3, T3, T4, and T7 SP fx | 5 | B | T3-7 posterior fusion | TBI/ICH, SDH, frontal/nasal facial fx, right rib fx 2-5, left rib fx 3-6, hemothorax, pulmonary contusion | 41 |
| 42 | F | 39.3 | - | 6 | 30 | 5 | Tibial plafond fx bilateral,  Talus fx, Tibial  plateau fx,  Knee dislocation | L: L3 burst fx | 7 | D | L2-4 posterior fusion | - | 32 |
| 19 | F | 23.9 | - | 9 | 11 | 5 | Ankle fx | TL: L2 burst fx,  L2 TP fx | 5 | D | T12-L4 posterior fusion, interbody | - | 20 |
| 28 | M | 25.7 | Tobacco | 9 | 25 | 4 | Tibial plafond fx | L: L3 burst fx | 7 | D | L1-L5 posterior fusion | - | 25 |
| 22 | F | 33.3 | - | 5 | 7 | 4 | - | TL: T12 burst fx | 7 | E | T10-L2 posterior fusion | - | 16 |
| 23 | F | 27.8 | - | 8 | 10 | 3 | - | TL: L2 flexion-distraction fx, L1 comp fx | 8 | D | T12-L4 posterior fusion | - | 16 |
| 39 | F | 25.4 | - | 6 | 20 | 4 | Tibial plafond fx | L: L3 burst fx, L2 SP fx | 7 | E | L1-5 posterior fusion | - | 25 |
| 39 | F | 32 | - | 9 | 43 | 23 | - | T: T8 flexion-distraction fx,  T6 comp fx,  T9 TP fx | 9 | A | T7-10 posterior fusion | TBI/ICH, IPH, SDH, SAH, subgaleal hematoma, scalp laceration, occipital and temporal bone fx, left 7-8 rib fx, hemothorax | 41 |
| 51 | F | 27.1 | - | 5 | 11 | 5 | - | TL: L1 burst fx,  T12 comp fx,  L2 comp fx | 5 | E | T11-L3 posterior fusion | - | 20 |
| 30 | M | 26.7 | - | 5 | 11 | 3 | Calcaneus fx,  Coccyx fx | TL/L: L4 burst fx,  L1 comp fx | 7 | D | L3-5 posterior fusion | - | 20 |
| 26 | F | 29 | Tobacco | 5 | 11 | 4 | - | TL: L1 flexion-distraction fx | 10 | E | T11-L3 posterior fusion | - | 16 |
| 41 | F | 29.1 | HTN | 3 | 7 | 2 | Midfoot fx | TL: L2 burst fx,  T12 comp fx,  L1 comp fx | 4 | D | T12-L4 posterior fusion | - | 24 |
| 53 | M | 30.5 | - | 3 | 11 | 2 | Pelvic ring fx | TL/L: L4 burst fx,  T12 comp fx,  L2 comp fx,  L3 comp fx | 5 | D | L2-S1 posterior fusion | - | 24 |
| 27 | F | 27.9 | - | 6 | 9 | 2 | Coccyx fx | TL/L: L1 burst fx,  L5 TP fx | 5 | E | T11-L3 posterior fusion | - | 20 |
| 21 | F | 28.7 | - | 5 | 10 | 3 | Perilunate dislocation,  Triquetral fx | TL/L: T12 burst fx,  T5 comp fx,  L1 TP fx | 8 | D | T10-L2 posterior fusion | - | 24 |
| 48 | M | 25.4 | - | 4 | 21 | 5 | Tibial plateau fx,  Radial distal fx | TL: L1 burst fx | 7 | D | T11-L3 posterior fusion | - | 25 |
| 40 | F | 33.6 | - | 8 | 23 | 5 | Pelvic ring fx | TL: T12 flexion-distraction fx | 10 | D | T10-L2 posterior fusion | - | 20 |

C: cervical (C1-7); DM2: diabetes mellitus type II; T: thoracic (T1-9); TL: thoracolumbar (T10-L2); L: lumbar (L3-5); TP: TP; SP: SP; comp: comp

HTN: hypertension; fx: fx; LOS: length of stay; ICU: intensive care unit

TBI: traumatic brain injury; ICH: intracranial hemorrhage; SDH: subdural hematoma; SAH; subarachnoid hemorrhage; IPH: intraparenchymal hemorrhage
